# Burden of migraine among Egyptian people: prevalence and comorbidities

**DOI:** 10.1186/s10194-025-02016-0

**Published:** 2025-05-13

**Authors:** Ahmed Amir Samir, Ahmed W. Hageen, Ahmed Elgammal, Mostafa Meshref, Mennatullah A. El-Refaay, Mohamed Medhat Taalap, Ali Elsaeed Nassef, Rawan Ali Bedewe, Ahmed Almeldein, Ibrahim Ali Kabbash

**Affiliations:** 1 Faculty of Medicine, Al-Azhar University, Cario, Egypt; 2 Faculty of Medicine, Tanta University, Tanta, Gharbia Egypt; 3 Department of Neurology, Faculty of Medicine, Al-Azhar University, Cairo, Egypt; 4 Faculty of Medicine, October’s 6 University, October’s 6, Egypt; 5 Faculty of Medicine, Alexandria University, Alexandria, Egypt; 6 Faculty of medicine, Assiut University, Assuit, Egypt; 7 Faculty of Medicine, Tanta University, Tanta, Gharbia Egypt

**Keywords:** Migraine, Depression, Anxiety, Insomnia, Disability, Egypt

## Abstract

**Introduction:**

Migraine is a prevalent debilitating neurological illness that stands among the top causes of disability and significantly impacts the quality of life. Migraine-related functional impairment involves physical, emotional, and economic consequences that frequently impact occupational, academic, social, and familial aspects of life. Depression, anxiety, and sleep disturbances are among the most common comorbid conditions associated with migraine.

**Objective:**

This study aimed to assess the prevalence of migraine among the Egyptian population and associated comorbidities.

**Methods:**

we conducted a cross-sectional study using a validated Arabic self-administered questionnaire distributed to the general population. The questionnaire was used to collect data on sociodemographic characteristics, migraine frequency, characteristic associated disability, insomnia, and psychological factors. Convenience snowball sampling method was utilized. Univariate and multivariate regression analyzes were applied.

**Results:**

A total of 2,533 participants were included in the final analysis from five Egyptian regions. Females represent 57%. More than one-half of participants (59.1%) aged 20–30 years. The prevalence of migraine was 20.9%. The most common triggers were sleep disorders (76.9%), followed by perceived noise (65%), and anxiety (59%). Among the participants diagnosed with migraine, 46.7% had a severe disability, 22% had clinical insomnia of moderate severity, 20.5% had severe depression, 29% had severe anxiety, and 20.6% had severe stress. Females, older age, and urban residents were the key predictors of migraine. Lifestyle factors including regular physical activity and good hydration were linked to reduced migraine risk. Comorbid conditions including insomnia, stress, and anxiety significantly impacted migraine severity.

**Conclusion:**

Our results showed a 20.9% prevalence of migraine, with nearly one-half of cases associated with severe disability, along with comorbidities like depression, anxiety, and insomnia. Female gender, older age, and urban residence are key predictors, while lifestyle factors such as physical exercise and good hydration reduce the risk of migraine.

**Supplementary Information:**

The online version contains supplementary material available at 10.1186/s10194-025-02016-0.

## Introduction

Headache is exceedingly common and can be classified as a debilitating medical condition that can lead to a diminished quality of life and disrupted productivity at work, eventually causing substantial economic hardship in societies [1]. Migraine is a type of primary headache disorder, characterized by recurrent attacks of throbbing headache that typically occur on one side of the head and are associated with other symptoms of neurological disorders with specific characteristics, such as phonophobia, photophobia, visual or sensory disturbances, nausea, and vomiting [1, 2]. These episodes are frequently brought out by stress, specific meals, weather, dehydration, coffee, changes in sleep habits, smoking, the use of medications, and sudden exposure to light or strong odors [3]. 

Migraine is a highly prevalent neurological disorder worldwide. It is ranked as the seventh health issue that results in the reduction of life expectancy due to disability [4]. Migraine stands as the second-leading etiology resulting in disability-adjusted life years (DALYs) lost globally [5]. Migraine prevalence ranges from 11 to 16% in females and 5–8% in males, 19% among university students with higher rates noticed among people aged 25–55 years [6, 7]. The prevalence of migraine is affected by gender (females are two to three times more likely than males), age, and appears to be influenced by genetic, regional, cultural, and environmental factors [8]. Migraine is more prevalent after adolescence, peak between the ages of 35 and 39, and subsequently tend to decline with age especially after menopause [9]. 

Migraine is a challenging neurological disorder that negatively impacts a persons’ ability to effectively perform their job or occupational performance, family, and education duties [10]. Compared to healthy individuals, people with migraine have a significantly lower quality of life, psychological, social, emotional, and physical well-being [11]. Moreover, migraine is significantly accompanied by high financial requirements, particularly due to indirect determinants including wastage of occupational time and reduced productivity [12]. 

Migraine is recognized as a debilitating condition that frequently coexists with other co-morbidities. Numerous mental health conditions have been linked to migraine, and there is growing research regarding the reciprocal relationship between migraine and psychiatric disorders [13]. Depression and anxiety are two of the most common comorbidity associated with migraine. Moreover, generalized anxiety disorder (GAD), obsessive-compulsive disorder (OCD), panic disorder, stress, bipolar disorder, and post-traumatic stress disorder are correlated in both directions, with one increasing the risk of the other [13, 14]. Prior research has demonstrated that migraine combined with anxiety and depression may increase the risk of chronic migraine, poor treatment, outcomes, and medical expenses [15]. In addition, increased migraine frequency was associated with higher scores for anxiety and depressed symptoms [16]. Furthermore, the relationship between migraine and sleep disorders is complex and bidirectional [17]. In addition to being a cause for migraine, sleep is a treatment for the condition [17, 18]. Additionally, studies reveal that sleep disturbances, particularly insomnia, are more prevalent in individuals who get migraine at regular intervals as well as they are the cause of migraine in 8.3 − 64% of cases [18, 19]. In addition to depression, anxiety, and sleep disorders, migraine is linked to several common neurological disorders such as stroke, epilepsy, and multiple sclerosis [20, 21]. 

Despite migraine therapy and knowledge having evolved significantly, migraine has tended to be under-diagnosed and under treated, specifically, migraine sufferers in low-resource environments still receive inadequate care [5]. Migraine is a prevalent primary headache in Egypt, affecting a large proportion of the population, especially females and individuals aged between 20 and 40 years. Studies showed a high prevalence 2800/100 000 in Al Quseir City, Red Sea Governorate [22], 17.3% in Fayoum Governorate [23], and 10.51% in Assiut Governorate [24]. 

Migraine is a prevalent yet under-recognized neurological disorder in Egypt, significantly impacting individuals’ quality of life and productivity. Despite its association with disability, insomnia, and psychological comorbidities, comprehensive data on its burden in Egypt remain scarce, necessitating further research. This study aims to bridge this gap by assessing the prevalence of migraine, its associated disability, and related comorbidities including insomnia and psychological distress.

## Materials and methods

### Study design, settings, and target population

Between August and December 2024, an observational cross-sectional study was conducted by using an anonymous, self-administration web-based survey that was distributed to the general population in all regions including Greater Cario, Alexandria, Delta, Upper Egypt, and the Suez Canal. Egyptians regardless of gender, aged 18 years or older, from any region, and willing to complete the survey in Arabic language were invited to complete the survey. Non-Egyptians, and individuals suspected of having a secondary cause of headache such as sinusitis, history of head trauma, and neurological disorders were excluded.

### Sampling and sample size calculation

Participants were recruited using a convenient snowball sampling method. Using the Epi Info statistical software 7.2.6. version, the sample size was calculated based on: a 95% confidence interval, a 5% acceptable margin of error, an expected frequency of 37% based on previous study [25], and design effect of 2.5% because of non-randomization of sample selection. The minimum sample size was 895 participants. As the study covers five regions and to increase the study’s power and ensure that all regions were included, the sample size was raised to **3687**.

### Data collection tools

Data for this research was collected using a structured, self-administered questionnaire. The questionnaire was adapted based on a previous Arabic-validated questionnaire. The questionnaire is depicted in Appendix 1. The questionnaire included a self-reported screening question at the beginning to identify potential participants with secondary causes of headache. The questionnaire was divided into five sections:

1. The first section included the participants’ sociodemographic and lifestyle characteristics
2. The second section encompassed the migraine screening questionnaire (MS-Q). MS-Q is a self-administered tool for migraine detection. The Arabic translation was validated by Alaqeel et al. with Cronbach alpha between 0.81 and 0.83 [26]. The MS-Q includes five questions about headache frequency, features, and the presence or absence of migraine-related symptoms. Each negative response (no) gets 0 points, while positive answers (yes) score one point. A score of four or more indicated migraine suspicion. In addition, a question regarding migraine triggers.
3. The third section utilizes the Migraine Disability Assessment Scale (MIDAS), which is used to assess the impact of migraine on productivity. To help Arabic-speaking patients manage their migraine better, a validated Arabic version of the MIDAS was developed. It showed good reliability with a Cronbach alpha of 0.812 [27]. It is consisting of five questions. The sum of these five questions forms the MIDAS score. The MIDAS sub-scale score is divided into: Grade I (0–5 = little or no disability), Grade II (6–10 = mild disability), Grade III (11–20 = moderate disability), and Grade IV (> 21 = severe disability).
4. The fourth section concluded the insomnia severity index (ISI). The ISI scale was translated and validated by Hallit et al. and showed a high Cronbach alpha of 0.833 [28]. It consists of seven questions based on DSM IV diagnostic criteria for insomnia. ISI sub-scale score is divided into: (0–7) no clinically significant insomnia, (8–14) subthreshold insomnia, (15–21) clinical insomnia of moderate severity, and (22–28) severe clinical insomnia.
5. The final section assesses mental health status by utilizing the Compound Depression, Anxiety, and Stress Scale (DASS-8). The Cronbach’s alpha for this scale is 0.94, demonstrating high reliability, and its validity was established by Ali et al. [29] It includes eight questions with responses zero for never, one for sometimes, two for often, and three for always. The total score of DASS-8 ranges between zero to 24 and subscale divided into depression, anxiety, and stress. The total depression score is divided into normal depression (0–3), moderate depression (4–6), and severe depression (7–9). Anxiety total score subdivided as depression score and stress subscale divided into normal stress (0–2), moderate stress (3–4), and severe stress (5–6).

### Pilot study

A pilot study was conducted to test reliability of the questionnaire. The survey was distributed to 45 individuals. The key goals of the pilot study were to determine the clarity, comprehensibility, and usefulness of the questionnaire items, as well as to detect any potential inconsistencies or cultural discrepancies. Participants were encouraged to provide feedback on the survey’s content, such as the clarity of the questions, the appropriateness of the language used, and the total length of the questionnaire. Based on the feedback received, modifications were made to improve clarity of some questions. The reliability and internal consistency of the survey were examined using Cronbach’s alpha, which was 0.71 for the MS-Q, 0.88 for MDAS, 0.81 for ISI, and 0.86 for DASS-8.

### Data collection method

An internet-based survey using the “Google Forms” platform was sent to the target population via various common social media platforms (Facebook, WhatsApp, Telegram, and X platform). We allocated 5–7 collaborators (Migraine group of Egypt) to each region to manage data collection and promote participation to achieve the target sample size. At the beginning of the survey, an overview of the study and objectives were provided as well as informed consent. To prevent duplicating data, IP addresses were activated in Google Forms. To avoid information bias, all participants who provided incomplete responses were excluded.

### Ethical considerations

The Declaration of Helsinki’s guiding principles were followed during the conduct of the study. Contribution to this study was voluntary. The participants’ identities and confidentiality were maintained during the study, involving data collection and processing. The ethical approval of the study was obtained from the Institutional Review Board (IRB) of the Faculty of Medicine, Tanta University, Tanta, Egypt (Approval number: 36264PR847/9/24).

### Statistical analysis

Statistical analysis was conducted using R statistical software version 4.3.1 (2023-06-16 ucrt). Categorical variables were summarized as frequency and percentage. Univariate and multivariate regression analysis were applied including sociodemographic characteristics as independent variables for the first model, lifestyle and health factors for the second model, and insomnia and mental health variables for the third model. The results were presented as odds ratio (OR) and 95% confidence interval (CI) with significance set at *p* < 0.05. Notably, only significant results of bivariate analysis were included in multivariable logistic regression models.

## Results

A total of 3.687 participants completed the survey, 1023 were excluded because they were incompatible with our eligibility criteria. Of them, 2.2% (*n* = 84) were non-Egyptian, 0.5% (*n* = 20) did not provide informed consent, and 27.2% (*n* = 1,003) were suspected to have secondary headache. A total of 2,533 participants were included in the final analysis.

### Demographic and lifestyle characteristics of the participants

A total of 2,533 participants were included in the final analysis. Their mean age was 23.91 + 6.62 and with a range of 18–68 years. Female participants represented 57%. More than one-half of the participants (59.1%) were aged 20–30 years and the majority were currently not married 85.7%). Monthly family income was reported as just enough by 48.1% and 62.7% were of urban residents. The Delta region had the highest rate of responses (33.6%). Among participants, 85.9% had a university education and 60.3% were working or studying in a medical field. Concerning employment status, university students represented 60.6%. (Table 1)

**Table 1:** Sociodemographic characteristics of study participants

Variables	Number (*n* = 2533)	%
**Gender**		
Female	1444	57.0
Male	1089	43.0
**Age (years)**		
< 20	752	29.7
20–30	1497	59.1
> 30	284	11.2
Range	18–68	
Mean *±* SD	23.91 + 6.62	
**Marital status**		
Currently not married	2172	85.7
Currently married	361	14.3
**Family Income**		
Not enough	629	24.8
Just enough	1219	48.1
More than enough	685	27.0
**Residence**		
Rural	945	37.3
Urban	1588	62.7
**Regions**		
Greater Cairo	564	22.3
Alexandria	324	12.8
Delta	851	33.6
Suez Canal	183	7.2
Upper Egypt	611	24.1
**Educational level**		
University	2177	85.9
Below University	356	14.1
**Field of study or work**		
Medical	1527	60.3
Non-medical	1006	39.7
**Employment status**		
University Student	1536	60.6
Farmer/Manual work	69	2.7
Office Work	208	8.2
Professional Work	590	23.3
Unemployed	130	5.1

**Table 2:** Llifestyle characteristics of study participants

Variables	Number (*n* = 2533)	%
**Physical activity (hours/ week)**		
No physical activity	784	31.0
Less than 1.5 h/w	517	20.4
1.5–3 h/w	503	19.9
4–5 h/w	364	14.4
More than 5 h	365	14.4
**Fluid intake (litre/day)**		
Less than 1 L	316	12.5
1–2 L	1432	56.5
3–4 L	647	25.5
More than 4 L	138	5.4
**Average sleep hours**		
6 h or less	787	31.1
7–8 h	1299	51.3
More than 8 h	447	17.6
**Smoking status**		
**No**	2364	93.3
**Yes**	169	6.7
**Daily caffeine intake**		
No	1163	45.9
Yes	1370	54.1
**Chronic disease**		
No	1724	68.1
Yes	809	31.9
**Family history of migraine**		
No	1914	75.6
Yes	619	24.4

Table (2) shows the lifestyle patterns of studied participants where 31.0% reported nonpractice of regular physical activities while only 14.4% had regular physical activity for more than five hours per week. Most participants (56.5%) reported an intake of 1–2 L of water per day. An average of 7–8 sleep hours was reported by 51.3% and 93.3% were non-smokers. More than one-half (54.1%) reported a daily intake of caffeine. Those with chronic diseases represented 31.9% and 24.4% reported a family history of migraine.

### Migraine prevalence, triggers, and associated severity

**Fig. 1:**
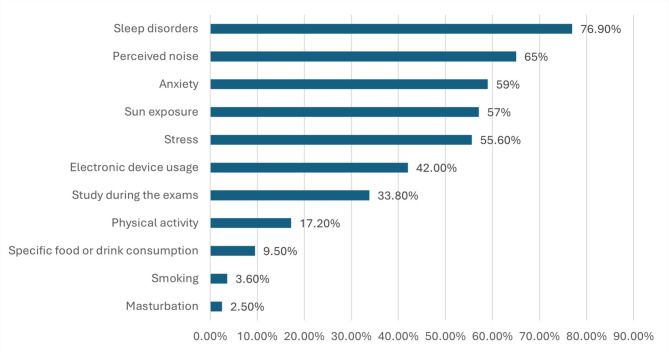
The percentage distribution of the main triggering factors of migraine

**Fig. 2:**
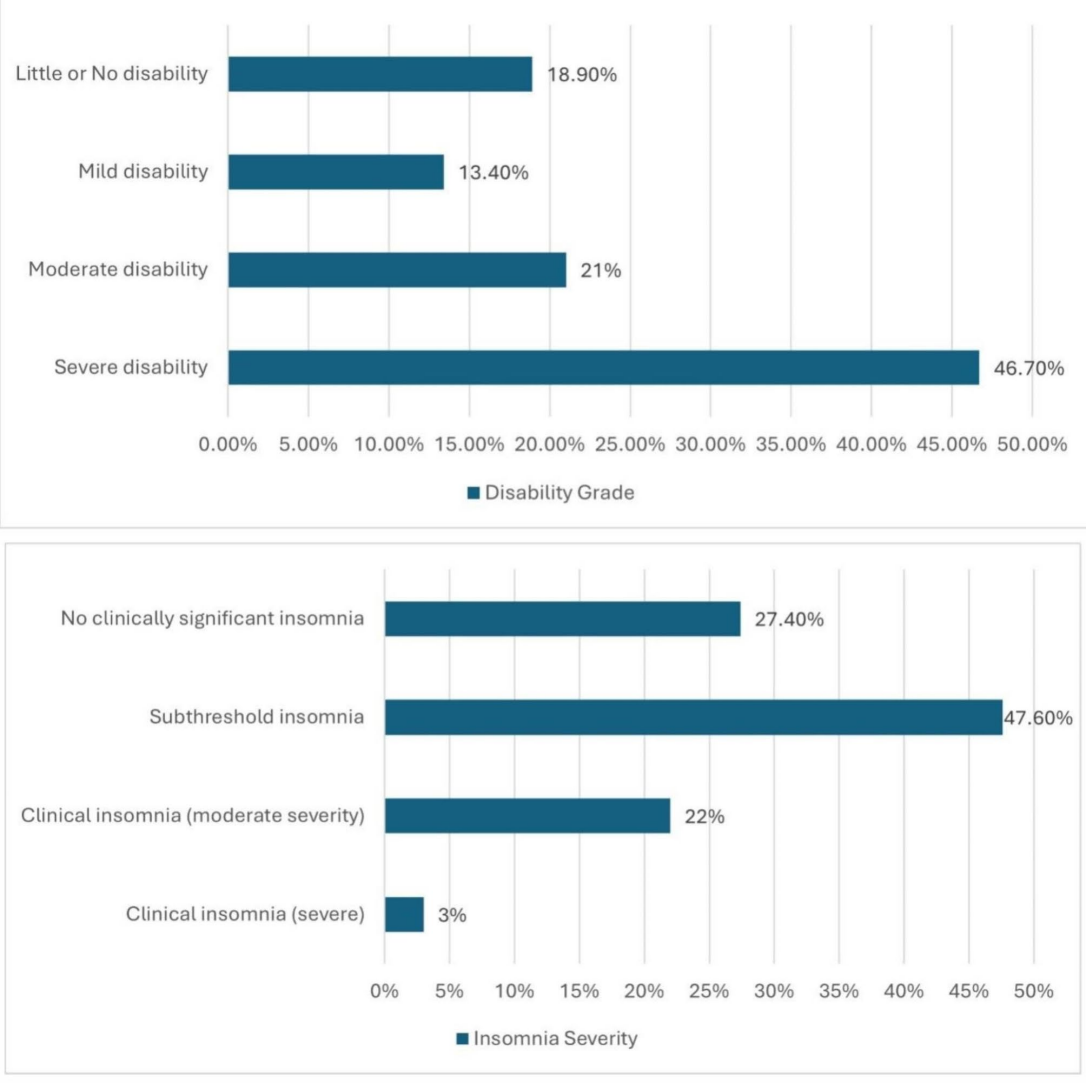
Distribution of disability and insomnia levels among participants diagnosed with migraine (*n* = 529)

**Fig. 3:**
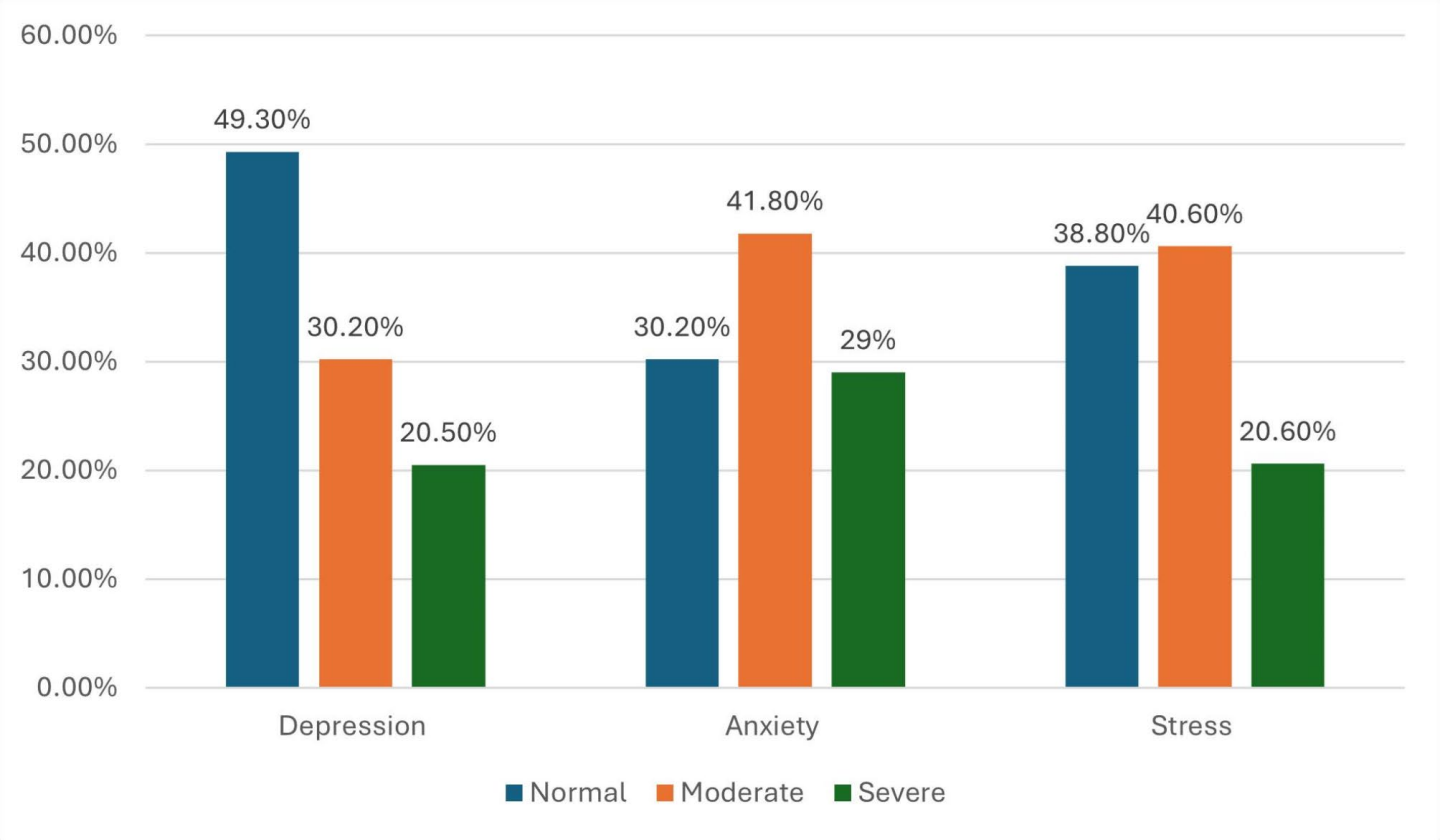
Distribution of depression, anxiety, and stress among participants diagnosed with migraine (*n* = 529)

A total of 2533 study participants were assessed for migraine using MS-Q. Of them, 20.9% had a score of ≥ 4, suggesting a likely diagnosis of migraine. For participants diagnosed with migraine, various potential migraine triggers were reported. The most common triggers were sleep disorders (76.9%), followed by perceived noise (65%), and anxiety (59.0%) (Fig. 1**).** Among the participants diagnosed with migraine, 46.7% had severe disability related to migraine, 22% had clinical insomnia (moderate severity), 20.5% had severe depression, 29% had severe anxiety, and 20.6% had severe stress. (Figs. 2 and 3)

### Associations between migraine, sociodemographic, and lifestyle characteristics of study participants

In the multivariate analysis, male participants had significantly lower odds of positive migraine screening (aOR = 0.30, 95% CI: 0.24–0.38, *p* < 0.0.01). Age over 30 years was associated with higher odds of migraine compared to those under 20 years (aOR = 1.79, 95% CI: 1.12–2.87), while no significant differences were observed for the 20–30 age group. Urban residents had increased odds of migraine (aOR = 1.33, 95% CI: 1.05–1.69) compared to rural residents. No associations were found for marital status, family income, region, educational level, field of study, or employment status. (Table 3)

**Table 3:** Sociodemographic characteristics of study participants and their associations with migraine

Variables	Migraine Screening				OR (95% CI, *P*-value) (univariate)	aOR (95% CI, *P*-value) (multivariate)
	Negative (*n* = 2004)		Positive (*n* = 529)			
	*n*	%	*n*	%		
**Gender**						
Female	1028	71.2	416	28.8	1	1
Male	976	89.6	113	10.4	0.29 (0.23–0.36)**	0.30 (0.24–0.38)**
**Age (years)**						
< 20	593	78.9	159	21.1	1	1
20–30	1213	81.0	284	19.0	0.87 (0.70–1.09)	0.95 (0.75–1.22)
> 30	198	69.7	86	30.3	1.62 (1.19–2.20)**	1.79 (1.12–2.87)*
**Marital status**						
Currently not married	1734	79.8	438	20.2	1	1
Currently married	270	74.8	91	25.2	1.33 (1.03–1.72)*	0.87 (0.58–1.29)
**Family Income**						
Not enough	499	79.3	130	20.7	1	
Just enough	971	79.7	248	20.3	0.98 (0.77–1.25)	Excluded #
More than enough	534	78.0	151	22.0	1.09 (0.83–1.41)	Excluded #
**Residence**						
Rural	793	83.9	152	16.1	1	1
Urban	1211	76.3	377	23.7	1.62 (1.32-2.00)**	1.33 (1.05–1.69)*
**Regions**						
Greater Cairo	406	72.0	158	28.0	1	1
Alexandria	253	78.0	71	21.9	0.72 (0.52–0.99)*	0.89 (0.63–1.25)
Delta	693	81.4	158	18.6	0.59 (0.46–0.75)**	0.95 (0.72–1.26)
Suez Canal	152	83.1	31	16.9	0.52 (0.34–0.79)**	0.69 (0.44–1.08)
Upper Egypt	500	81.8	111	18.2	0.57 (0.43–0.75)**	0.80 (0.59–1.08)
**Educational level**						
University or above	1713	78.7	464	21.3	1	
Below University	291	81.7	65	18.3	0.82 (0.61–1.09)	Excluded #
**Field of study or Work**						
Medical	1223	80.1	304	19.9	1	
Non-medical	781	77.6	225	22.4	1.16 (0.95–1.41)	Excluded#
**Employment status**						
University Student	1236	80.5	300	19.5	1	1
Farmer/Manual work	63	91.3	6	8.7	0.39 (0.15–0.84)*	0.58 (0.24–1.41)
Office Work	167	80.3	41	19.7	1.01 (0.70–1.44)	1.1 (0.71–1.70)
Professional Work	445	75.4	145	24.6	1.34 (1.07–1.68)*	1.29 (0.98–1.7)
Unemployed	93	71.5	37	28.5	1.64 (1.09–2.43)*	1.15 (0.73–1.81)

* *P* value significance ≤ 0.05.**P value < 0.01, Excluded# for being insignificant in bivariate analysis

Abbreviations: OR (Odds ratio), aOR (adjusted odds ratio), CI (Confidence interval)

The multivariate analysis showed that those with regular physical activity for 1.5–3 h/week were significantly less likely to suffer from migraine compared to those with no physical activity (aOR0.72, 95%CI = 0.53–0.97). Increased fluid intake was associated with a statistically significant decreased risk for migraine, as the adjusted OR was 0.7 for those reporting fluid intake of 1–2 L /day and decreased to reach 0.5 among those reporting drinking more than four liters. Participants who reported sleeping for 7–8 h were significantly less likely to suffer from migraine than those sleeping less or more (0.66, 95% CI = 0.52–0.82). Daily intake of caffeine increased the risk of migraine where the adjusted OR was 1.39 (95% CI = 1.13–1.70). History of chronic disease increased the risk of migraine by 2.12 (95% CI = 1.73–2.60). A positive family history of migraine significantly increased the risk of suffering from migraine with an adjusted OR of 2.20 (95% CI = 1.78–2072). (Table 4)

**Table 4:** Llifestyle characteristics of study participants and their associations with migraine

Variables	Migraine Screening				OR (95% CI, *P*-value) (univariate)	aOR (95% CI, multivariate)
	Negative (*n* = 2004)		Positive (*n* = 529)			
	*n*	%	*n*	%		
**Physical activity**						
No physical activity	599	76.4	185	23.6	1	1
Less than 1.5 h/w	391	75.6	126	24.4	1.04 (0.80–1.35)	1.1 (0.84–1.45)
1.5–3 h/w	418	83.1	85	16.9	0.66 (0.49–0.87)**	0.72 (0.53–0.97)*
4–5 h/w	295	81.0	69	19.0	0.76 (0.55–1.03)	0.82 (0.59–1.13)
More than 5 h	301	82.5	64	17.5	0.69 (0.50–0.94)*	0.79 (0.56–1.10)
**Fluid intake (litre)**						
Less than 1 L	224	70.9	92	29.1	1	1
1–2 L	1127	78.7	305	21.3	0.66 (0.50–0.87)**	0.71 (0.53–0.94)*
3–4 L	536	82.8	111	17.2	0.50 (0.37–0.69)**	0.63 (0.45–0.88)**
More than 4 L	117	84.8	21	15.2	0.44 (0.25–0.73)**	0.5 (0.29–0.86)*
**Average sleep hours**						
6 h or less	581	73.8	206	26.2	1	1
7–8 h	1074	82.7	225	17.3	0.59 (0.48–0.73)**	0.66 (0.52–0.82)**
More than 8 h	349	78.1	98	21.9	0.79 (0.60–1.04)	0.87 (0.65–1.16)
**Smoking status**						
No	1863	78.8	501	21.2	1	
Yes	141	83.4	28	16.6	0.74 (0.48–1.10)	Excluded#
**Daily caffeine intake**						
No	959	82.5	204	17.5	1	-
Yes	1045	76.3	325	23.7	1.46 (1.20–1.78)**	1.39 (1.13–1.7)**
**Chronic disease**						
No	1450	84.1	274	15.9	1	1
Yes	554	68.5	255	31.5	2.44 (2.00-2.97)**	2.12 (1.73–2.60)**
**Family history of migraine**						
No	1591	83.1	323	16.9	1	1
Yes	413	66.7	206	33.3	2.46 (2.00-3.02)**	2.2 (1.78–2.72)**

* *P* value significance ≤ 0.05.**P value < 0.01, Excluded# for being insignificant in bivariate analysis

Abbreviations: h/w (hours/ week), OR (Odds ratio), aOR (adjusted odds ratio), CI (Confidence interval)

### Associations between migraine, insomnia, depression, anxiety, and stress

The different levels of severity of insomnia were significantly associated with migraine as compared to no clinically significant insomnia. The adjusted OR was 2.4 for sub-threshold insomnia, 3.89 for moderate insomnia, and increased to 4.36 for severe insomnia (95% CI = 1.89–3.05, 2.79–5.43, and 1.97–9.64, respectively). The level of depression was not found to independently affect the risk of migraine. Meanwhile, the adjusted OR for severe anxiety as a risk of migraine was 1.80 (95% CI = 1.24–2.60, *p* < 0.01). In addition, the adjusted OR for moderate and severe stress was associated with an increased risk for migraine by 1.62 and 2.35, respectively (95% CI = 1.24–2.12 and 1.56–3.53, respectively). (Table 5)

**Table 5:** Migraine among study participants and their associations with participants’ insomnia, depression, anxiety, and stress levels (*n* = 2533)

Variables	Migraine Screening				OR (95% CI, *P*-value) (univariate)	aOR (95% CI, multivariate)
	Negative		Positive			
	*n*	%	*n*	%		
**Insomnia Severity**						
No clinically significant insomnia	1190	89.1	145	10.9	1	1
Subthreshold insomnia	660	72.4	252	27.6	3.13 (2.51–3.93)**	2.4 (1.89–3.05)**
Moderate severity	141	54.9	116	45.1	6.75 (5.00-9.12)**	3.89 (2.79–5.43)**
Severe	13	44.8	16	55.2	10.10 (4.77–1.78)**	4.36 (1.97–9.64)**
**Depression Score**						
Normal	1444	84.7	261	15.3	1	1
Moderate	429	70.1	183	29.9	2.36 (1.90–2.93)**	1.05 (0.8–1.37)
Severe	131	60.6	85	39.4	3.59 (2.65–4.85)**	0.86 (0.56–1.33)
**Anxiety Score**						
Normal	1123	87.5	160	12.5	1	1
Moderate	668	75.6	216	24.4	2.27 (1.81–2.85)**	1.3 (0.99–1.7)
Severe	213	58.2	153	41.8	5.04 (3.87–6.58)**	1.8 (1.24–2.6)**
**Stress Score**						
Normal	1350	86.8	205	13.2	1	1
Moderate	514	70.5	215	29.5	2.75 (2.22–3.42)**	1.62 (1.24–2.12)**
Severe	140	56.2	109	43.8	5.13 (3.83–6.85)**	2.35 (1.56–3.53)**

* *P* value significance ≤ 0.05. **P value < 0.01

*Abbreviations: OR (Odds ratio), aOR (adjusted odds ratio), CI (Confidence interval)

## Discussion

**M**igraine is a prevalent neurological disorder, recognized for its significant public health implications. Migraine is estimated to impact 15% of the total global population [30], while in Middle Eastern countries, the prevalence ranges from 2.6 to 32% [31]. However, the incidence varied significantly by country: 22.5% in Saudi Arabia [32], 7.7% in Jordan [33], 30.8% in Morocco [34], 23.1% in Kuwait [35], 16.4% in Turkey [36], 14.3% in United Kingdom [37], 22.8% in India [38], and 11.7% in United States [9], and 15.1% in Iran [39]. The variation in migraine prevalence may be explained by genetic or environmental factors and socioeconomic conditions. Moreover, our population-based study estimated a prevalence rate of 20.9%. However, other studies among Egyptians reported a prevalence of 2.8% for those aged more than 8 years in Al Quseir City, Red Sea Governorate [22], 17. 3% among people aged 15–83 years in Fayoum Governorate [23], and 10.55% among the population in Assiut governorate [24]. The alignment with previous studies suggests that the burden of migraine is significant in both local and broader regional contexts.

Consistent with previous studies in Egypt [22–24], our study revealed that females have a higher incidence of migraine than males. This is consistent with global trends indicating that migraine is more common in women than in men. Previous literature implies a gender ratio is two to three times more common in women than in men [40]. This gender difference is possibly explained by hormonal effects, different pain responses, differences in structure and function in specific brain areas, potential genetic factors, as well as behavioral and dietary habits. Estrogens have a significant impact on neuro excitability, shape, and function of specific brain areas, which may explain why women are more prone to migraine than men [41]. 

Migraine typically begin during adolescence and affect people aged 35 to 45 in general [1]. In 2021, 35-39-year-olds had the highest prevalence of migraine [42]. Moreover, in the ME region, migraine mainly impact people in their early to mid-adult years, as the mean patient age varied from 27 to 37.5 years [31]. Aligning with these findings, our results showed that age over 30 years was associated with higher odds of migraine compared to those under 20 years. A study in Egypt showed peak migraine prevalence among 20-40-year-olds [24]. However, multiple studies reported the migraine prevalence to be in the teens or early twenties [43, 44]. This difference may be due to the characteristics of the studied sample and demographic factors. This age range is crucial for social interactions, job advancement, and educational success. The present study reported that urban residents have increased migraine occurrence compared to rural residents. Similarly, these results were also reported in previous studies [45, 46]. This is possibly due to the multiple stressors that face people from urban areas. As well as environmental factors and lifestyle differences which contribute to migraine incidence.

As regards the lifestyle factors, participants with regular physical activity for 1.5–3 h/week are significantly less likely to suffer from migraine compared to those with no physical activity. Multiple studies noticed that regular physical activity lowers migraine prevalence and frequency [47–49]. Exercise may stimulates endogenous neurotransmitter signals that may effectively diminish the intensity of migraine pain [50]. Increased fluid intake was associated with a statistically significant decreased risk for migraine. This association was also demonstrated in the study of Khorsha F, et al. [51] Another study reported that dehydration and decreased water intake may increase the migraine occurrence [52]. Participants with good sleeping periods (7–8 h) were significantly less likely to suffer from migraine than those sleeping less or more. This agreed with a previous study reporting that reduced sleep duration (< 6 h per day) correlates with elevated episodes of headache in individuals with migraine [53]. 

For many years, caffeine has been associated with migraine, both as a trigger and a cure [54]. Our study showed that daily intake of caffeine was associated with an increased risk of migraine episodes. Similarly, a previous study reported that the risk of migraine headache increased with a caffeine intake of ≥ 400 mg/day. However, patients with migraine should be aware of the amount of caffeine they consume and not exceed 200 mg daily [54]. Previous research data indicated that the specific effects of caffeine on sleep, cerebral circulation, and intracranial pressure can vary depending on when consumed during the sleep-wake cycle. There may be a great deal of individual variation in caffeine metabolism, which affects the therapeutic and/or harmful effects of caffeine [55]. However, there is not enough data to suggest that all patients with migraine should stop using caffeine; nevertheless, it should be pointed out that excessive caffeine consumption may trigger migraine cornification, and abrupt withdrawal from caffeine can precipitate migraine attacks [54]. 

History of chronic disease increases the risk of migraine. Previous studies mentioned the increased risk of migraine episodes in patients with hypertension or diabetes [56, 57]. This might be due to the affection of those chronic diseases on the cerebral circulation. A positive family history of migraine significantly increases the risk of migraine. This went with previous studies that discussed the family history of migraine and the development of episodic or transformation into chronic migraine [58, 59]. There is a significant hereditary component to migraine although no pattern of inheritance has been found. Relatives of affected persons are three times more likely to get migraine than relatives of unaffected individuals [60]. Our study demonstrated that different lifestyle factors including physical activity, hydration, sleep quality, and caffeine intake significantly influence migraine prevalence. This urges for the importance of promoting healthy habits and tailored interventions to mitigate migraine risk effectively.

For participants diagnosed with migraine, various potential migraine triggers were reported. The most common triggers were sleep disorders, followed by perceived noise, and anxiety. Similarly, among Middle Eastern countries, the most common triggers of migraine were sleep disorders, dietary habits, and stress [31]. Migraine triggers are quite heterogeneous, offering insights into brain functioning. From atmospheric changes to hormones to foods, they initiate brain pathways and migraine symptoms [61]. Such multifactorial migraine triggers need to be approached holistically and as one single factor in disrupting the recurrence cycle. On the other hand, recent data pointed out the even bigger importance of balance in trigger avoidance with building resilience, for example, stress-coping mechanisms [62]. Future research should focus on refined personalized prevention protocols. Understanding these triggers is crucial for the effective management and treatment of migraine.

Various comorbidities were associated with migraine including depression, anxiety, stress, and insomnia. Similar to our finding, these comorbidities were reported among ME countries [31]. Severe depression occurred in 20.5% of participants with migraine. This aligned with a previous meta-analysis which reported that the prevalence range of depression among patients with migraine ranged from 8.6 to 47.9% [63]. Another study reported the prevalence rate of depression among migraine patients at 42.6% that ranged from mild to severe forms [64]. The occurrence of depression among patients with migraine may be due to decreased levels of 5-hydroxytryptamine (5-HT), or serotonin receptors. Also, there was evidence of serotonin transporter gene alterations which increased the risk of depression and migraine occurrence [65]. In the present study, 29% of patients with migraine had severe anxiety. These results agreed with the previously published meta-analysis which reported the prevalence of anxiety among patients with migraine ranging from 16 to 83% [66]. In addition, a previous study reported that mood and anxiety disorders are two to ten times more common in patients with migraine than in the general population [13]. This may be attributed to the worrying process and increased concern of having attacks of migraine between patients with migraine [67]. Severe stress occurred in 20.6% of migraine patients participating in the present study. A review in ME showed that stress is the most frequently listed cause by migraine sufferers as a trigger for their attacks [31]. Stress can trigger the onset of migraine, exacerbate migraine-related disabilities and burdens, and lead to the progression of chronic forms of migraine. Furthermore, migraine attacks and their associated disability may act as stressors, creating a vicious cycle [68]. Thus, these findings emphasize the complex relationship between migraine and a variety of other health issues.

Individuals experiencing migraine are considerably more prone to inadequate sleep quality and nocturnal exhaustion [33]. There is a complicated and reciprocal relationship between headache and sleep disturbances [17]. Moreover, insomnia is by far the most prevalent sleep disturbance among headache sufferers [17, 69]. Among our participants 22% had clinical insomnia (ranging from subthreshold to moderate and severe). This observation corresponded with the findings reported by Kim et al., which indicated a greater prevalence of insomnia in migraine patients; approximately 25.9% [9]. Consistent with our findings, a review article revealed that sleep disturbances induce both migraine and tension headache and that insomnia is more common in both migraine and non-migraine headache sufferers [70]. Insomnia reduces both mental and physical performance and is associated with a variety of daily life deficits [71]. Our findings demonstrated that different levels of severity of insomnia were significantly associated with migraine. This was discussed in the study of Waliszewska-Prosół M. and his colleagues who reported that any changes in sleep duration, sleep quality, and associated fatigue may contribute to increased headache [71]. Similarly, other studies reported the same results [72, 73]. 

Migraine significantly impacts disability, functional limitations, and psychological impacts. Most clinicians fail to recognize the severity and extent of migraine-induced impairment, leading to a reduced quality of life and disability [74]. Among the participants diagnosed with migraine in our study, 46.7% had a severe disability related to migraine. This result is close to the result of the previous study of Al Ghadeer HA, et al., who reported severe disability in 57.3% of participants [74]. Consequently, these disabilities substantially affect patients’ physical and emotional health, resulting in impairments and decreased productivity [31]. 90% of participants in a randomized clinical trial in the United States showed that migraine attacks made living more difficult and caused disability. Approximately 68% reported that their social and familial lives as well as their productivity at work were affected [75]. Patients with higher monthly headache frequency had more activity impairment and more frequent visits to health care providers, according to a cross-sectional survey carried out in five European countries [76]. 

### Implications for public health and clinical practice

To reduce stigma and enhance early migraine management, awareness programs about migraine symptoms and its comorbidities should be implemented to educate the public. Customize migraine treatment plans for different populations, paying special attention to urban dwellers and women of childbearing age. In addition to routine migraine assessment, mental health screenings should be applied to identify and manage comorbid conditions effectively. Promoting lifestyle modifications like regular physical activity and good hydration through awareness campaigns. In addition to modification of lifestyle, train the patients in the management of their special triggers through personalized action plans such as stress management techniques. Regular follow-ups should assess treatment efficacy and adherence. The long-term effects of lifestyle modifications on migraine frequency and intensity, as well as the efficacy of new treatment modalities in varying populations, should continue to be investigated. Further longitudinal studies to explore the causal relationships between migraine and their comorbidities can inform future therapeutic strategies.

### Strengths and limitations

Our study is characterized by distinct strengths. The large sample size from various Egyptian regions improved the study’s findings’ generalizability. We investigated a wide range of factors including different sociodemographic, lifestyle, and psychological factors to understand the migraine’s multifaceted impacts on health. As well as its related disability and associated comorbidities. Furthermore, a significant relationship between migraine and depression, anxiety, and insomnia, contributes to developing a more profound comprehension of the intricate interplay between these conditions which impact quality of life. Also, use well-structured and validated questionnaires to cover all variables measured. Despite these strengths, our study does have some limitations. Even though there were notable significant associations between the variables under investigation, the cross-sectional nature makes it difficult to draw definitive conclusions on the direction of the associations between migraine and the factors under evaluation. Future longitudinal designs can more accurately evaluate all these issues. Self-reported survey-based questionnaires are prone to recall biases. Convenience and snowball sampling techniques may have resulted in selection bias. Our data may not have accurately represented all age groups due to differences in electronic media use among the population. Another limitation of our study is the demographic characteristics of the sample, which was predominantly female, urban, and comprised mostly of university students. This specific demographic may limit the generalizability of the findings to the broader Egyptian population. Finally, migraine diagnosis was based on MS-Q rather than clinical diagnosis, potentially leading to overestimation or misclassification of migraine cases. Further studies should screen migraine incidence by clinical evaluation.

## Conclusion and recommendations

Our finding demonstrated a 20.9% prevalence of migraine. Migraine was found to be associated with severe disability in nearly one-half of cases in addition to insomnia and depression. The study also revealed migraine predictors that include female gender, old age, and urban residence besides some lifestyle factors that reduced the risk of migraine including physical exercise and good hydration. Therefore, there is a need for specific public health strategies pertaining both to the physical and psychological dimensions of migraine treatment. To reduce healthcare costs, multidisciplinary management plans should be implemented including behavior and lifestyle modification, pharmacological interventions, patient education, and routine screening for the coexistence of migraine with psychiatric disorders.

## Electronic supplementary material

Below is the link to the electronic supplementary material.

Supplementary Material 1

## Data Availability

Data is available at the reasonable request of the corresponding author.

## Abbreviations

DALYs: Disability-adjusted life years
GAD: Generalized anxiety disorder
OCD: Obsessive-compulsive disorder
MS-Q: Migraine screening questionnaire
MIDAS: Migraine Disability Assessment Scale
ISI: Insomnia severity index
DASS-8: Compound depression, anxiety, and stress scale
OR: Odds ratio
CI: Confidence interval
ME: Middle East
5-HT: 5-hydroxytryptamine
