# Supplementary material for: Burden of migraine among Egyptian people: prevalence and comorbidities
Source: J Headache Pain. 2025 May 13;26(1):114. doi: 10.1186/s10194-025-02016-0 (PMC12070771; doi:10.1186/s10194-025-02016-0)
Supplement: Supplementary file 1 — Supplementary Material 1 [file 10194_2025_2016_MOESM1_ESM.docx]

**Supplemental Appendix**

**Burden of Migraine among Egyptian People: Prevalence and Comorbidities**

**Supplemental Appendix Contents**

**Table S1.** The questionnaires

| **Informed Consent**  This is a cross-sectional study aimed at assessing the prevalence of migraine among the Egyptian population and studying its impact on mental health and quality of life.  The survey will take 10-15 minutes. No personal information will be collected or contacted. The information you provide during the survey will be used for a research project that will be published later. If you agree to participate, please click “I agree.”  Thank you for your time. |
| --- |
| **Have you been diagnosed with one of these diseases or exposed to such problem?** |
| - Hypertension - Hydrocephalus - Brain hemorrhage - Any tumor in the brain - Any operation in the head - Head injury - Chronic Sinusitis - No, I haven't been diagnosed or had any of these problems |
| **Section 1: Sociodemographic and Lifestyle Characteristics** |
| 1. **Gender** |
| - Female /Male |
| 1. **Age (years): ……** |
| 1. **Marital status** |
| - Currently married / Currently not married |
| 1. **Family Income** |
| - Not enough / Just enough/ More than enough |
| 1. **Residence** |
| - Rural / Urban |
| 1. **Regions** |
| - Greater Cairo / Alexandria/ Delta/ Suez Canal/ Upper Egypt |
| 1. **Educational level** |
| - University / Below University |
| 1. **Field of Study or work** |
| - Medical / non-medical |
| 1. **Employment status** |
| - University Student/ Farmer and/or Manual work/ Office Work / Professional Work/ Unemployed |
| 1. **How many hours a week do you do physical activity, such as walking, swimming, cycling, strength training such as lifting weights, or other physical activity?** |
| - No physical activity/ Less than 1.5 h/w/ 1.5-3 h/w/ 4-5 h/w/ More than 5 h |
| 1. **How much fluid do you drink on a moderate temperature day؟** |
| - Less than 1 L/1-2 L/3-4 L/ More than 4 L |
| 1. **Average number of hours of sleep per day?** |
| - 6 hours or less/ 7-8 hours/ More than 8 hours |
| 1. **Are you smoke?** |
| - No / Yes |
| 1. **Do you drink coffee daily؟** |
| - No / Yes |
| 1. **Do you have a positive Family history of migraine** |
| - No / Yes |
| 1. **Do you suffer from any chronic diseases?** |
| - No / Yes |
| **Section 2: Migraine screening questionnaire (MS-Q) and migraine triggers** |
| 1. **Do you have frequent or intense headaches** |
| - No / Yes |
| 1. **Do your headaches usually last more than 4 hours** |
| - No / Yes |
| 1. **Do you usually suffer from nausea when you have a headache** |
| - No /Yes |
| 1. **Does light or noise bother you when you have a headache** |
| - No / Yes |
| 1. **Does headache limit any of your physical or intellectual activities** |
| - No / Yes |
| **Which of the following triggers your headache (You can choose more than one answer)?** |
| - Noise |
| - Anxiety sleeping disturbance |
| - Physical activity |
| - Exposure to sun |
| - Studying for exams |
| - Stress |
| - Electronic device use |
| - Eating habits |
| - Menstruations |
| - Smoking |
| **Section 3: Migraine disability assessment scale (MIDAS)** |
| **Please answer the following questions about all the headaches you have had over the last 3 months. Select your answer in the box next to each question. Select zero if you did not have the activity in the last 3 months.** |
| 1. On how many days in the last 3 months did you miss work or school because of your headaches? |
| 1. How many days in the last 3 months was your productivity at work or school reduced by half or more because of your headaches? (Do not include days you counted in question 1 were you missed work or school.) |
| 1. On how many days in the last 3 months did you not do household work (such as housework, home repairs and maintenance, shopping, caring for children and relatives) because of your headaches? |
| 1. How many days in the last 3 months was your productivity in household work reduced by half of more because of your headaches? (Do not include days you counted in question 3 where you did not do household work.) |
| 1. On how many days in the last 3 months did you miss family, social or leisure activities because of your headaches? |
| **Section 4: Insomnia severity index (ISI)** |
| **For each question, please CIRCLE the number that best describes your answer.** |
| 1. **Difficulty falling asleep** |
| - None - Mild - Moderate - Severe - Very Severe |
| 1. **Difficulty staying asleep** |
| - None - Mild - Moderate - Severe - Very Severe |
| 1. **Problems waking up too early** |
| - None - Mild - Moderate - Severe - Very Severe |
| 1. **How SATISFIED/DISSATISFIED are you with your CURRENT sleep pattern?** |
| - Very Satisfied - Satisfied - Moderately Satisfied - Dissatisfied - Very Dissatisfied |
| 1. **How NOTICEABLE to others do you think your sleep problem is in terms of impairing the quality of your life?** |
| - Not at all Noticeable - A Little – Somewhat -Much - Very Much Noticeable |
| 1. **How WORRIED/DISTRESSED are you about your current sleep problem?** |
| - Not at all worried - A Little – Somewhat -Much - Very Much Noticeable |
| 1. **To what extent do you consider your sleep problem to INTERFERE with your daily functioning (e.g. daytime** 2. **fatigue, mood, ability to function at work/daily chores, concentration, memory, mood, etc.) CURRENTLY?** |
| - **Not at all Interfering - A Little – Somewhat -Much - Very Much Noticeable** |
| **Section 5: Depression, Anxiety and Stress Scale.** |
| Please read each statement and circle a choice which indicates how much the statement applied to you over the past week. There are no right or wrong answers. Do not spend too much time on any statement. |
| 1. **I felt that I had nothing to look forward to** |
| - Did not apply to me at all |
| - Applied to me to some degree, or some of the time |
| - Applied to me to a considerable degree, or a good part of time |
| - Applied to me very much, or most of the time |
| 1. **I felt downhearted and blue.** |
| - Did not apply to me at all |
| - Applied to me to some degree, or some of the time |
| - Applied to me to a considerable degree, or a good part of time |
| - Applied to me very much, or most of the time |
| 1. **I was unable to become enthusiastic about anything.** |
| - Did not apply to me at all |
| - Applied to me to some degree, or some of the time |
| - Applied to me to a considerable degree, or a good part of time |
| - Applied to me very much, or most of the time |
| 1. **I was worried about the situations in Which I might panic and make a fool of myself.** |
| - Did not apply to me at all |
| - Applied to me to some degree, or some of the time |
| - Applied to me to a considerable degree, or a good part of time |
| - Applied to me very much, or most of the time |
| 1. **I felt I was close to panic.** |
| - Did not apply to me at all |
| - Applied to me to some degree, or some of the time |
| - Applied to me to a considerable degree, or a good part of time |
| - Applied to me very much, or most of the time |
| 1. **I felt scared without any good reason** |
| - Did not apply to me at all |
| - Applied to me to some degree, or some of the time |
| - Applied to me to a considerable degree, or a good part of time |
| - Applied to me very much, or most of the time |
| 1. **I found it difficult to relax.** |
| - Did not apply to me at all |
| - Applied to me to some degree, or some of the time |
| - Applied to me to a considerable degree, or a good part of time |
| - Applied to me very much, or most of the time |
| 1. **I felt that I was using a lot of nervous.** |
| - Did not apply to me at all |
| - Applied to me to some degree, or some of the time |
| - Applied to me to a considerable degree, or a good part of time |
| - Applied to me very much, or most of the time |
